# Outcomes of transcorneal electrical stimulation therapy in the early stages of retinitis pigmentosa

**DOI:** 10.55730/1300-0144.5368

**Published:** 2022-01-23

**Authors:** M. Necati DEMİR, Uğur ACAR, Güngör SOBACI, Dinçer GÖKSÜLÜK

**Affiliations:** 1 Private Eye Clinic, Ankara, Turkey; 2 Department of Ophthalmology, Selçuk University Faculty of Medicine, Konya, Turkey; 3 Department of Ophthalmology, Kırıkkale University Faculty of Medicine, Kırıkkale, Turkey; 4 Department of Biostatistics, Erciyes University Faculty of Medicine, Kayseri, Turkey

**Keywords:** Multifocal electroretinography, retinitis pigmentosa, tes therapy, transcorneal electrical stimulation, visual field

## Abstract

**Background/aim:**

To investigate the effect of transcorneal electrical stimulation (TES) therapy in patients with retinitis pigmentosa (RP).

**Materials and methods:**

We performed TES therapy in 21 patients with RP in 12 sessions with 1-week intervals. The following parameters obtained before and after the TES therapy were compared statistically; the best corrected visual acuity (BCVA, logMAR), Ishihara color vision level, multifocal electroretinography (mf-ERG) response, automated visual field (VF) outcome, and the 25-item low vision quality-of-life (LVQOL) questionnaire points.

**Results:**

The mean age of patients (6 females; 15 males) was 31.67 ± 9.80 years (20–50 years). While increases in BCVA level, color vision level, mf-ERG response in p1 amplitude of ring 1, and LVQOL questionnaire points were statistically significant, changes in VF test and other mf-ERG responses were not. Twenty of the patients (95.24%) stated that they were satisfied with the TES therapy. No considerable side effect was observed in any patient due to the therapy.

**Conclusion:**

The TES therapy may be an effective and safe treatment modality in slowing the RP progression, especially in the early stages of the disease. Longer-term follow-ups in larger patient populations are warranted.

## 1. Introduction

Retinitis pigmentosa (RP) is a genetic retinal disorder which results from progressive degeneration of retinal photoreceptor layer and adjacent tissues [1]. In the early stages of disorder, patients with RP complain about night blindness only. As the disease progresses, the patients complain of narrowing of the visual field (VF) because of loss of the rod photoreceptor cells in the peripheral retina, and decreased vision in dim environments. It eventually leads to permanent blindness as the central vision is also affected. Numerous studies including stem cell and gene therapies, are currently performed under investigation for the treatment of RP, but there is no definitive treatment yet. There is not even a treatment method that ceased the vision loss in these patients destined to blindness.

Transcorneal electrical stimulation (TES) therapy is a newer treatment method that seems applicable to retinal diseases that have no cure in the current. TES therapy has been shown to slow the progression of RP and also leads to clinical improvement thanks to neuroprotective effects on the retina [2,3]. It is thought that TES application affects the remaining healthy retinal cells (dormant cells) [3]. There are also some case reports in the literature about the positive effects of TES therapy in some ocular pathologies with no definitive treatment [4–6].

We aimed to investigate the effects of TES therapy in RP patients in the early stages of disease in this study.

## 2. Materials and methods

During May 2017 to June 2018 period, the files of 21 RP patients who underwent TES therapy were analyzed. All participants had night vision problems associated with classical fundus findings including pallor optic disc, narrowed vascular tree, and bone-spicule pigmentation.

### 2.1. Inclusion criteria

1. Patients who had the typical clinical and electrophysiological findings of RP.
2. Age ≥20 years and ≤50 years
3. BCVA ≥ 0.1 (Snellen chart).
4. Patients who had TES therapy regularly for 12 sessions with a 1-week interval.
5. Patients who signed informed consent documents with sufficient understanding after receiving an explanation for the responsibility of TES therapies.
6. Patients with complete information on objective and subjective variables in their files.

### 2.2. Exclusion criteria

1. Patients who had any systemic disease.
2. Patients who had another anterior or posterior segment pathology
3. Patients who had an ocular trauma, or an eye operation except for cataract surgery.
4. Age <20 years or >50 years
5. BCVA < 0.1 (Snellen chart).
6. Patients who had advanced RP findings (severe optic disc atrophy, bone spicules inside the arcades, or macular pathology) according to Smith et al.’s grading clinical score for RP [7].
7. Patients who had the therapies at irregular intervals or incompletely.
8. Patients who do not have a written consent form in their file.

We performed TES therapy in RP patients with ocuvision system (CE approved, GmbH, Reutlingen, Germany) consisting of stimulating device (Ocustim), application spectacle (Ocuspex), and electrode (OcuEl) as described previously [2,3,8]. While an ocular electrode was placed on the cornea, two skin electrodes were placed on temple area bilaterally. After determining the value of electrical phosphene threshold (EPT), 12 sessions of TES therapy were performed on the patients with an interval of one week with the following parameters; 200% EPT, 200–400 μA power, 20 Hz frequency, 2 msec biphasic and 30 min duration.

All patients underwent a detailed ophthalmic examination before starting TES therapy and after the last TES therapy. The following parameters were compared 1 week before starting therapy and 1 week after all therapy has been completed; the BCVA (Snellen chart), Ishihara color vision level (the number of color plates reads correctly), multifocal electroretinography (mf-ERG; RetiScan 3.22.0.1; Roland Instruments, Wiesbaden, Germany) p1 wave amplitudes of ring 1, mean deviation (MD) in automated VF (24-2 SITA-SAP, Humphrey Field Analyzer II, Carl Zeiss Meditec, Inc., Dublin, CA, USA), the low vision quality-of-life (LVQOL) questionnaire points [9]. This questionnaire has 25 items, 11 subscales including overall activities, difficulty with near and distance vision activities, limitations in social functioning, dependency on others, mental health symptoms, driving difficulties, limitations with peripheral and color vision, ocular pain, and an additional subscale for general health. It has been created to measure the vision targeted health status for patients with chronic eye diseases causing low vision [9]. The mf-ERG responses of the patients with RP were recorded using special electrodes and a standard protocol as explained in detail previously [10]. The mf-ERG stimuli location and anatomical areas corresponded as follows: Ring 1, central hexagon overlying the fovea; Ring 2, the parafoveal area; Ring 3, the perifoveal area; Rings 4 and 5, the far peripheral retina. Measurements of rings 4 and 5 were not evaluated because they do not correspond to either fovea or peri/parafoveal area.

All ophthalmic examinations and LVQOL questionnaire were performed by the same ophthalmologist (MND), and all procedures were also performed under his supervision. Written informed consent was obtained from all individual participants, and all procedures were conducted according to the Declaration of Helsinki. This study was approved by the Review Board of Selçuk University Faculty of Medicine (2020/456).

### 2.3. Statistical analysis

For all analyses, the IBM-SPSS version 21.0 was used. The variables were described as mean ± standard deviation (SD). The effectiveness of TES therapy was evaluated by testing the mean differences against zero. For each numeric variable, mean differences were calculated by subtracting before TES therapy measurements from after TES therapy measurements. For statistical analysis, BCVA obtained with Snellen chart was converted to logMAR (logarithm of the minimum angle of resolution). Normality assumption was checked by Shapiro-Wilk’s test. Normally distributed differences were tested with one-sample t-test while nonnormally distributed differences were tested with one-sample Wilcoxon signed-rank test. Furthermore, categorical variables were reported using frequencies and percentages. A value of p < 0.05 was considered statistically significant.

## 3. Results

A total of 42 eyes of 21 patients with RP were included in the study. The mean age of patients (6 females; 15 males) was 31.67 ± 9.80 years (20–50 years). After the TES therapy statistically significant changes were determined in all parameters except improvements in VF test and mf-ERG responses other than p1 amplitude (Table 1, 2, and Figure).

All patients except one (95.24%) stated that they were satisfied with the TES therapy. The procedures were well tolerated by all participants. Trivial symptoms such as foreign body sensation, burning, and itching were observed in only 3 (14.29%) patients. All symptoms were transient and no considerable side effect and/or discomfort caused to cease the therapy in any patient.

## 4. Discussion

Although many different therapies have been applied to the treatment of RP, there is no definitive treatment of the disease yet [1,11–13]. This study showed that TES therapy provided positive objective and subjective outcomes on patients with RP. Especially, improvement in the LVQOL questionnaire and the high satisfaction rate were very gratifying and promising.

Morimoto et al. have shown that TES has a neuroprotective effect by some experimental studies [14–17]. Firstly, in 2002, Morimoto et al. [14] discovered that optic nerve stimulation with electricity increased the axotomized retinal ganglion cells (RGCs) survive. Then, the same authors indicated that TES provides to survive the axotomized RGCs by increasing IGF-1 levels [15]. Afterward, they investigated the TES effects in different gene models for RP, and they demonstrated that TES enabled photoreceptors to survive and preserved retinal function in RCS rats and in rhodopsin P347L transgenic rabbits [16,17]. Ni et al. [18] determined an upregulation of B-cell lymphoma 2 (Bcl-2), ciliary nerve trophic factor (CNTF), and brain-derived neurotrophic factor BDNF and a downregulation of Bcl-2 associated x protein (Bax) in the rats with light-induced photoreceptor degeneration. The authors also found that Bcl-2 and CNTF were selectively upregulated in Müller cells. Although the exact mechanism of the TES has not been identified, it is thought that the protective effects of therapy can be related with upregulation of some neurotrophic factors, or vasodilatory, antiapoptotic, antiglutamate, and antiinflammatory mechanisms [19,20].

The safety and the feasibility of TES therapy in patients with various ocular disorders such as RP, glaucoma, amblyopia, homonymous VF loss, and normal individuals were first investigated by Gekeler et al. [8]. They found that the TES therapy using DTL electrodes was safe, fast, and reliable.

There are four clinical studies evaluating the effects of TES therapy [2,3,21,22], and there is one study investigating the effects of transdermal electrical stimulation (TdES) [23] on RP patients in the literature. The efficacy and the safety of TES therapy in RP patients were first investigated by Schatz et al. with a prospective, randomized and sham-controlled clinical trial [2]. They performed TES therapy using DTL electrodes to 24 RP patients for 30 min for 6 consecutive weeks with a one-week interval. They divided patients into 3 groups: sham, 66%, or 150% of individual EPT. They determined statistically significant improvement in the VF and scotopic b-wave amplitude in 150% group, whereas no change was observed in 66% group. They reported foreign body sensation in only 2 (8.33%) patients as an adverse event as in our study. Afterward, the same authors designed a clinical trial with a larger and over longer period of time [3]. They performed the TES therapy on 52 RP patients for 30 min per week for 52 consecutive weeks. They divided patients into 3 groups: sham, 150%, or 200% of individual EPT. They found a significant improvement of cone function (light-adapted single flash b-wave amplitude) in both of the 150% and 200% EPT groups, and a significant improvement of rode function (scotopic b-wave amplitude) in 200% EPT group compared to the sham group.

We set the EPT value in our study as 200% in the light of these studies. We selected the RP patients who had early stages according to Smith et al.’s grading system considering 4 criteria for RP staging; lens status, optic disc appearance, the extent of bone spicule pigmentation and presence of macular pathology, developed in 2013 [7]. We included the patients who had BCVA of more than 0.1. We thought that these patients at this stage and BCVA level could have more dormant cells to be activated and respond psychophysical tests affirmatively.

Schatz et al. [2] performed TES therapy under the supervision of an investigator and an assisting nurse by using DTL electrodes in their first study, whereas it was performed at home conditions by patients and/or relatives themselves by using ocuvision system in their second study [3]. They declared that the OkuStim devices allow application of TES therapy by the patients themselves at home, these devices can be detected ineffective electrode positions, and they did not report any conspicuity in patients’ files pointing to mal- or dysfunction. In our study, the same ophthalmologist performed the procedure in each TES therapy and he has never left any patient alone and he continuously confirmed the contact between the electrode and the cornea. According to our observations; in the case of absolute interruption status, the device stops automatically, whereas the device can continue to run even if the contact between the electrode and the cornea decreases. Therefore, we do not find it appropriate to implement this therapy at home conditions.

Wagner et al. [21] reported a study investigating the safety and efficacy of TES treatment. They performed TES to 14 RP patients weekly for 30 min for 6 months under the supervision with 150% individual phosphene threshold. The authors also observed the participants for a further 6 months without any treatment. They did not detect any significant changes in the treatment group in terms of the visual acuity, microperimetry, Goldmann VF, optical coherence tomography and fundus autofluorescence outcomes compared to the control group. They also reported transient and spontaneous resolving foreign body sensation in 2 participants (14.28%), and discomfort underneath the skin electrode in 1 participant (7.14%) as the adverse events of TES therapy [21].

Kahraman and Oner [22] recently reported a prospective controlled study evaluating the safety and efficacy of TES treatment in RP patients. They compared BCVA, VF, and mfERG findings of 101 RP patients who underwent TES treatment 30 min once a week for 8 consecutive weeks and 100 RP patients who were enrolled as control. They determined a statistically significant but transient improvements in the treatment group. Since our study had a short follow-up time period, we may encounter such a negative result in the future. They did not observe any serious ocular side effects related to the TES therapy as in our study [22].

Miura et al. [23] evaluated the safety and efficacy of TdES therapy with skin electrodes in 20 eyes of 10 patients with RP. They performed the TdES 6 times at 2-week intervals with the following parameters; 1.0 mA power, 20 Hz frequency, 10 msec biphasic, and 30 min. They observed a statistically significant improvement in the mean BCVA level, and MD of the 10.2 Humphrey VF. No adverse events related to TdES were reported [23].

Our study results seem to be familiar to studies reported by Schatz et al. [2,3] and Kahraman and Oner [22], whereas it differed from Wagner et al’s study [21], considering the improvements in BCVA as well as color vision, p1 in mfERG and LVQOL. The fact that the number of patients in Wagner et al’s study was limited, applied EPT value was low, the mean age of patients was high (47.64 ± 18.76 years), and 5 (35.71%) of 14 participants had mild epiretinal membrane and 1 patient had staphyloma. In contrast to their study, we have achieved better objective and subjective improvements in RP patients.

Increased color vision, visual acuity, and p1 amplitude in mfERG suggest that TES therapy has positive effects on cone photoreceptors. We think that the more successful outcomes can be obtained by performing the TES therapy at an earlier stage of the disease. Our participants, compared to patients in other studies, might have more dormant cells. In our study, patients with BCVA lower than 0.1 level (Snellen) and severe RP findings were excluded from the study. Initial BCVAs of patients were better, and they were younger than the above-mentioned studies. Therefore, starting the therapy in the early stages with relatively preserved macula would result in more benefit from TES therapy. Wang et al. [24] also demonstrated that glucose replacement restored the dormant cone electrophysiology in a pig model of autosomal-dominant RP.

There are some limitations in our study. The number of our participants was limited as the patients who had the opportunity to apply this therapy in 12 sessions with 1-week intervals were included in the study. Our study also has a retrospective design and short follow-up time, and has not a control or sham group. Taking the BCVA from the patients with limited visual acuity such as RP would be better by using ETDRS chart which we do not have instead of Snellen chart. We were also able to define the molecular genetic basis in some of the participants only; therefore, we could not include this variable in the study. On the other hand, improvements in the LVQOL points together with safety of the procedures favor the use of TES therapy in RP patients.

The device used during the TES therapy also has some limitations such as the cost of the device and the kit being high, the absence of metal frames of different sizes for patients with different anatomy.

In conclusion; we found that TES therapy was effective, safe, and well-tolerated in patients with RP. Further studies with a larger number of patients exploring the optimum TES therapy parameters such as treatment dose, duration, session, EPT value, and the patient group that will benefit most from the treatment, are needed. We should continue to follow up the patients who underwent TES therapy in the long-term period in order to compare the long-term outcomes, too.

## Figures and Tables

**Figure:**
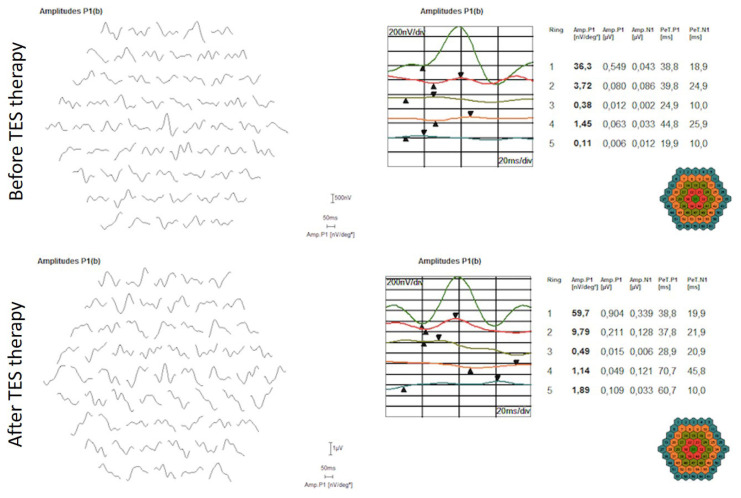
Multifocal electroretinography response of a patient with retinitis pigmentosa before and after TES therapy.

**Table 1:** Changes in the objective and subjective parameters before and after transcorneal electrical stimulation therapy.

	The mean ± standard deviation		P-value
	Before TES therapy	After TES therapy	
Best corrected visual acuity (LogMAR)	0.40 ± 0.31	0.26 ± 0.25	<0.001
Ishihara color vision level (plates)	12.48 ± 9.15	14.17 ± 8.27	<0.001
Mean deviation level in the automated visual field (dB)	−24.81 ± 6.68	−24.64 ± 6.83	0.31
Low vision quality-of-life questionnaire (points)	75.71 ± 17.11	88.90 ± 16.03	<0.001
Mean central foveal thickness (μm)	266.10 ± 50.95	265.95 ± 49.10	0.805

**Table 2:** Multifocal electroretinography responses of participants before and after TES therapy.

	The mean ± standard deviation		P-value
	Before TES therapy	After TES therapy	
p1 amplitude of ring 1 (nv/deg^2^)	38.32 ± 20.22	48.23 ± 22.00	<0.001
p1 implicit time of ring 1 (ms)	43.20 ± 10.81	39.89 ± 10.60	0.111
p1 amplitude of ring 2 (nv/deg^2^)	12.56 ± 4.08	12.99 ± 4.46	0.231
p1 implicit time of ring 2 (ms)	44.38 ± 11.29	41.48 ± 10.38	0.109
p1 amplitude of ring 3 (nv/deg^2^)	6.54 ± 3.46	7.18 ± 3.95	0.159
p1 implicit time of ring 3 (ms)	42.72 ± 10.04	41.26 ± 10.63	0.129
